# Exploration of visual analogue scale versions with the EQ-HWB-9 to measure health, social care, and carer-related quality of life: a VASt landscape

**DOI:** 10.1007/s11136-026-04369-8

**Published:** 2026-08-07

**Authors:** Jonathan L. Nazari, Maja Kuharic, Justin Yu, A. Simon Pickard

**Affiliations:** 1 Department of Pharmacy Systems, Outcomes and Policy, University of Illinois Chicago Retzky College of Pharmacy, Chicago, IL USA; 2 Department of Medical Social Sciences, Northwestern University Feinberg School of Medicine, Chicago, IL USA

**Keywords:** EQ-HWB, Health and wellbeing, Quality of life, Visual analogue scale

## Abstract

**Purpose:**

Visual analog scales (VAS) serve as brief, informative patient reported outcome measures. The EQ VAS is an important component of EQ-5D instruments but is not currently included in the EQ-HWB-9. This study qualitatively assessed perceptions of alternative VAS constructs and recall periods for potential inclusion alongside the EQ-HWB-9 and quantitatively examined VAS ratings.

**Methods:**

US adults who were: (1) diagnosed with chronic illness; (2) unpaid caregivers; (3) social care users (e.g., received caregiving, living with disability) completed an online survey and interview where they assessed VAS versions with health, health and wellbeing (HWB), and/or quality of life (QoL), as the specified construct. Participants discussed construct interpretation, appropriateness of recall periods (none, today, past 7 days), and whether these influenced their responses. Interviews were analyzed qualitatively through thematic analysis.

**Results:**

Among 34 participants, mean (SD) responses for health, HWB, and QoL were 61.9 (SD = 21.3), 60.9 (SD = 21.8) and 66.4 (SD = 21.5), respectively. All three VAS items were strongly correlated (*r* = 0.84–0.96). Interpretations of health focused on clinical, physical health more than mental/emotional health, HWB indicated mental/emotional health, and QoL evoked broader assessments encompassing finances or social-relationships. Most participants preferred having no specified recall period, allowing consideration of timeframes relevant to their experiences.

**Conclusion:**

Although quantitative results suggested that the constructs of health, HWB, and QoL were similar, qualitative results found that they were perceived differently, highlighting the importance of a mixed-methods approach. Further research in larger samples and other countries could help to inform the extent to which constructs and recall periods affect VAS responses.

**Supplementary Information:**

The online version contains supplementary material available at https://doi.org/10.1007/s11136-026-04369-8.

## Introduction

Rating scales, including visual analogue scales (VAS), are brief, informative methods to measure and value health outcomes on a continuous scale [1, 2]. A VAS can provide interval‑level data to complement an instrument’s descriptive system, either by measuring a different construct or by anchoring the same construct on a continuous metric. Existing EQ-5D instruments include an accompanying VAS, the EQ VAS, which asks respondents to rate their current health status on a vertical line ranging from 0 to 100, anchored with the “worst imaginable” and “best imaginable” health [3]. However, a VAS was not included in the development framework of the EQ-HWB-9, a generic, preference-based measure designed to evaluate health, social care, and carer-related quality of life [4, 5]. 

While initially conceived as a means to prime respondents for valuation tasks rather than measure individual health status, the EQ VAS has become an important complement to the EQ-5D descriptive system and the health state utility values that are determined based on population-level scoring through value sets [6, 7]. The EQ VAS does not have the same limitations as the EQ-5D index score (reflecting population-level preferences) and exists as a truer self-assessment that can inform patient-centered decision making [6, 8]. Although the EQ-5D is less commonly used in regulatory submissions to the European Medicines Agency (EMA) or US Food and Drug Administration (FDA), where it typically plays a supplementary role, EQ VAS ratings can be especially useful when index values are not easily interpretable or when patient-centered assessments are prioritized, including in health technology assessment (HTA) contexts. In one review of uses of the EQ-5D in HTA, the EQ VAS was used in nearly 70% of HTAs that included the EQ-5D, particularly in Germany where patient-centered outcomes are prioritized [6]. Research applications of the EQ VAS show that it broadens respondents’ considerations of their health status beyond the EQ-5D items, particularly incorporating broader conceptions of mental health [9, 10]. 

Thus, a VAS alongside the EQ-HWB-9 could be an important and valuable addition to the instrument, with potential roles as a valuation task, a summary of the descriptive system on a single, continuous metric, or a complement of the instrument’s nine items and utility estimates with a patient-centered global assessment. The use-cases of a VAS alongside the EQ-HWB-9 by researchers and policymakers are dependent on design decisions, including related to the specified construct and recall period, and their associated measurement properties. There are ongoing uncertainties about what the EQ VAS actually measures, how design features such as labels and endpoints influence interpretation, and how respondents conceptualize their responses [3, 7, 11]. Additionally, the construct validity of the EQ VAS has been found to vary based on mode of administration (self-complete or interview), population type (general public or patients), and geographic region (generally better construct validity in Europe and North America versus Asia) [3]. While incorporating the existing EQ VAS within the EQ-HWB-9 would confer an advantage of comparability with EQ-5D instruments, other options could be considered to either address shortcomings of the EQ VAS and/or tailor the VAS to align with the broader measurement focus of the EQ-HWB-9.

There are several conceivable advantages to having a VAS component with the EQ-HWB-9 in the US context, justifying it as the setting for initial evidence to inform instrument development. US English is the second most frequently requested EQ-5D version, and thus a large potential market for the EQ-HWB-9. With the role of patient reported outcomes increasing in importance in the US, including by the US FDA and Patient Centered Outcomes Research Institute (PCORI), it is essential for instrument development to include US-based testing and validation. In addition, the ‘Protecting Health Care for All Patients Act’ of 2022 stipulates that quality-adjusted life years (QALYs) should not be used in value-assessments for federal programs [12]. As the EQ-5D is closely associated with the use of QALYs [13], an accompanying VAS with the EQ-HWB-9 may offer additional value to decisionmakers within the US.

The primary aim of this study was to qualitatively assess US participant perceptions of several versions of a VAS, with variations in the construct and recall period, for potential inclusion alongside the EQ-HWB-9: specifically, how participants interpret different constructs (health, quality of life, health and wellbeing) and recall periods (today, past 7 days, or none) when completing a VAS. The secondary aim was to gather preliminary quantitative VAS response data to guide development of further quantitative studies to test VAS versions. These insights are intended to contribute to ongoing scientific and methodological discussions about how best to conceptualize and implement a VAS within the EQ-HWB-9.

## Methods

### Recruitment

Participants were purposively recruited through ResearchMatch (a national health volunteer registry created by several academic institutions and supported by the US National Institutes of Health Clinical Translational Science Award (CTSA) program, which has a large population of volunteers who have consented to be contacted by researchers about health studies for which they may be eligible). Potential participants were screened based on information available within ResearchMatch and contacted via email to confirm eligibility. Eligible participants were English-speaking, US adults (ages ≥ 18) meeting one or more of the following criteria, aligning with target populations for the EQ-HWB-9:

1. Diagnosed chronic illness–defined as any chronic physical or mental illness, impairment, or disability diagnosed by a healthcare provider and persisting for at least 12 months;
2. Current role as an unpaid caregiver – defined as individuals who reported providing unpaid, informal help (including helping with personal needs, medical needs, household chores, finances, arranging for outside services, or regularly visiting) to an adult (e.g., a friend or family member) either living with them or not living with them who was sick, disabled, or elderly;
3. Social care use –defined as individuals reporting that they received support to help them function in their daily life over the past 12 months, including paid or unpaid caregiving, home healthcare, living with a disability, residing in a care facility, or receiving assistance with personal care, home, or medical transportation needs.

The target sample size was approximately 7–10 participants from each of the three target groups, as recommended by the Consensus-based Standards for the selection of health Measurement Instruments (COSMIN) standards for content validity of patient-reported outcome measures [14]. The exact sample size was determined by qualitative saturation of themes [15], which was monitored throughout the interview process and determined to be reached when no new themes or insights emerged from successive interviews, indicating that the sample was sufficient to capture the range of experiences and perspectives relevant to the research question.

## Survey and interview elements: measures, design and justifications

### The EQ-HWB-9

The EQ-HWB is an experimental, generic measure of health, social care, and carer-related quality of life, developed through global qualitative and quantitative assessments encompassing input from individuals with physical and/or mental health conditions, social care recipients, and their caregivers [4, 16, 17]. The EQ-HWB-9 is the nine-item short form intended as a preference-based measure. Items include Daily Activities, Mobility, Exhaustion, Loneliness, Cognition, Anxiety, Sadness/Depression, Control, and Pain, each with a 7-day recall period and five-level frequency, severity, or difficulty response scale. Unlike EQ-5D instruments, the EQ-HWB-9 does not currently include an accompanying VAS.

### VAS versions

The EQ VAS construct (“health”), recall period (“today”), and anchors (worst/best imaginable) were used as a starting point for the VAS versions tested alongside the EQ-HWB-9, with the rationale that the EQ VAS would be a viable option with a strong, existing evidence base. In terms of testing specified constructs, Health can be defined broadly (for example, by the World Health Organization), so testing the construct “health” was considered a useful benchmark to compare against alternatives. Related, potentially broader constructs identified for testing included “health and wellbeing” (as in the name of the instrument, EQ-HWB) and “quality of life” (a familiar term aligned with the overarching construct intended to be measured by these instruments, health-related quality of life). Similarly, options considered for a specified recall period included “today” (as in the EQ VAS), the “last 7 days” (as in the EQ-HWB-9, maintaining internal consistency with the instrument’s items) or with no specified time frame, reflecting that broader constructs may not be meaningfully measured in a fixed window (as used, for example, in physical function items in Patient Reported Outcome Measurement Information System (PROMIS)) [18]. Anchor wording was deliberately held constant so that differences in participants’ interpretations could be attributed to the construct and the recall period only.

With three variations each in construct and recall period, a total of nine versions were evaluated. Each VAS version was presented with the selected construct and recall period to be measured on a vertical, 0-100 scale anchored from “the worst [construct] you can imagine” to “the best [construct] you can imagine” (shown in supplementary material).

### Survey procedures

Interested individuals were emailed an online Qualtrics survey where they provided sociodemographic and health information, confirming their eligibility criteria. Respondents completed the EQ-HWB-9 and three VAS versions in a random order, each without a specified recall period. After concluding the survey, respondents were invited to schedule a follow-up interview. To reduce fraudulent responses, unique survey links were sent, and survey responses were verified against the information available in ResearchMatch profiles.

### Interview procedures

A structured interview guide and accompanying slides were developed, informed by the COSMIN framework for assessing content validity [14] and previous EQ-HWB content validation studies [19, 20]. The interview materials were pilot tested in two recorded interviews with PhD students unaffiliated with the project acting as participants. These interviews were observed by the full research team and informed minor revisions to the interview materials to improve clarity and flow.

One-on-one, semi-structured interviews were conducted online via Zoom following a three-step test interview format including think aloud exercises, focused questions, and semi-structured discussion [21]. All interviews were conducted by JLN, the lead author. JLN had previous qualitative research experience and a clinical background in pharmacy providing direct patient care for a range of chronic health conditions. No prior relationship existed between the interviewer and participants excepting email communications to schedule appointments.

The interviewer initiated each session by reviewing the study purpose and procedures before obtaining verbal informed consent, including permission to record. During the interview, participants were shown visual representations of the three VAS versions without a recall period, as they had completed in the survey. Participants responded to structured prompts regarding how they did (or would) interpret and approach each version. Considering one VAS version at a time, participants were asked to reflect on the meaning of each construct and how these considerations influenced their rating. Participants were also asked which constructs aligned or diverged with the items that comprised the EQ-HWB-9. Once each construct had been discussed, participants were directed to consider whether they had thought of a particular recall period or timeframe when completing the VAS versions. Participants were then shown the same VAS versions with the recall period specified as today or the past 7 days and asked to comment on how a specified recall period may change their impression of the version and subsequent response. Finally, participants were asked to consider each possible VAS option holistically in terms of construct and recall period, and comment on which version they felt would be best to include alongside the EQ-HWB-9. Interviews also included discussions of the content validity of the modified EQ-HWB-9 items (published separately [22]), either prior to or after the discussion of the VAS items.

Interviews were approximately 60–90 min in duration, with approximately 15–30 min devoted to discussing the VAS. Participants received a $50 digital gift card as compensation. Immediately following each interview, the interviewer recorded brief field notes to document context and impressions.

### Data analysis

For quantitative data from the survey, descriptive statistics (means, standard deviations), effect sizes (Cohen’s d), correlations (Pearson’s r), and agreement (intraclass correlation coefficient (ICC)) were computed across the VAS variations.

Audio recorded interviews were transcribed verbatim and de-identified by the research team, using the Zoom automatic transcriptions as a starting point. Audio files to validate the transcripts were stored securely, accessible only to the research team. Transcripts were analyzed using the framework method, a type of content analysis where inductive and deductive methods are utilized to derive a working analytical framework of codes to compare data across and within individual transcripts [23, 24]. Coders familiarized themselves with the content of the transcript, noting initial impressions and referencing notes made during the interview. They independently read the transcripts line-by-line, identifying striking features in the data. An initial coding framework was developed deductively related to understanding of each construct, understanding of each recall period, preference for each construct and recall period, and alignment with the EQ-HWB, and refined iteratively to establish codes, including those inductively derived by classifying themes within the identified text. To ensure consistency, three transcripts were dual coded by two members of the research team (MK, JY) and discussed among the team to establish consensus. The remaining transcripts were coded by one team member (JLN, MK, JY) and reviewed by JLN or MK. The research team met to refine code definitions and organize codes into a structured framework.

Exemplary quotes were populated into an analytic framework and progress was discussed by the research team during weekly meetings. Inductive thematic saturation was monitored throughout the study through ongoing review of coded transcripts and was considered achieved when no new codes or concepts emerged in the interviews [15]. The analytical framework was finalized after all transcripts were coded. No participants reviewed transcripts or study findings. All analysis was conducted in Microsoft Word and Excel (Microsoft Corporation, Redmond, WA).

### Ethical considerations

The study received ethics approval from the University of Illinois Chicago (IRB# 2025 − 0169). Informed consent was obtained electronically via the pre-interview survey, and verbal consent was re-confirmed prior to the interview. Participants provided explicit permission for audio recording. A distress protocol was in place in the event of participant discomfort during the interview; however, no such incidents occurred. The qualitative portion of the study followed the Consolidated Criteria for Reporting Qualitative Research (COREQ) checklist, included in supplemental materials [25]. 

## Results

### Participant characteristics

**Fig. 1:**
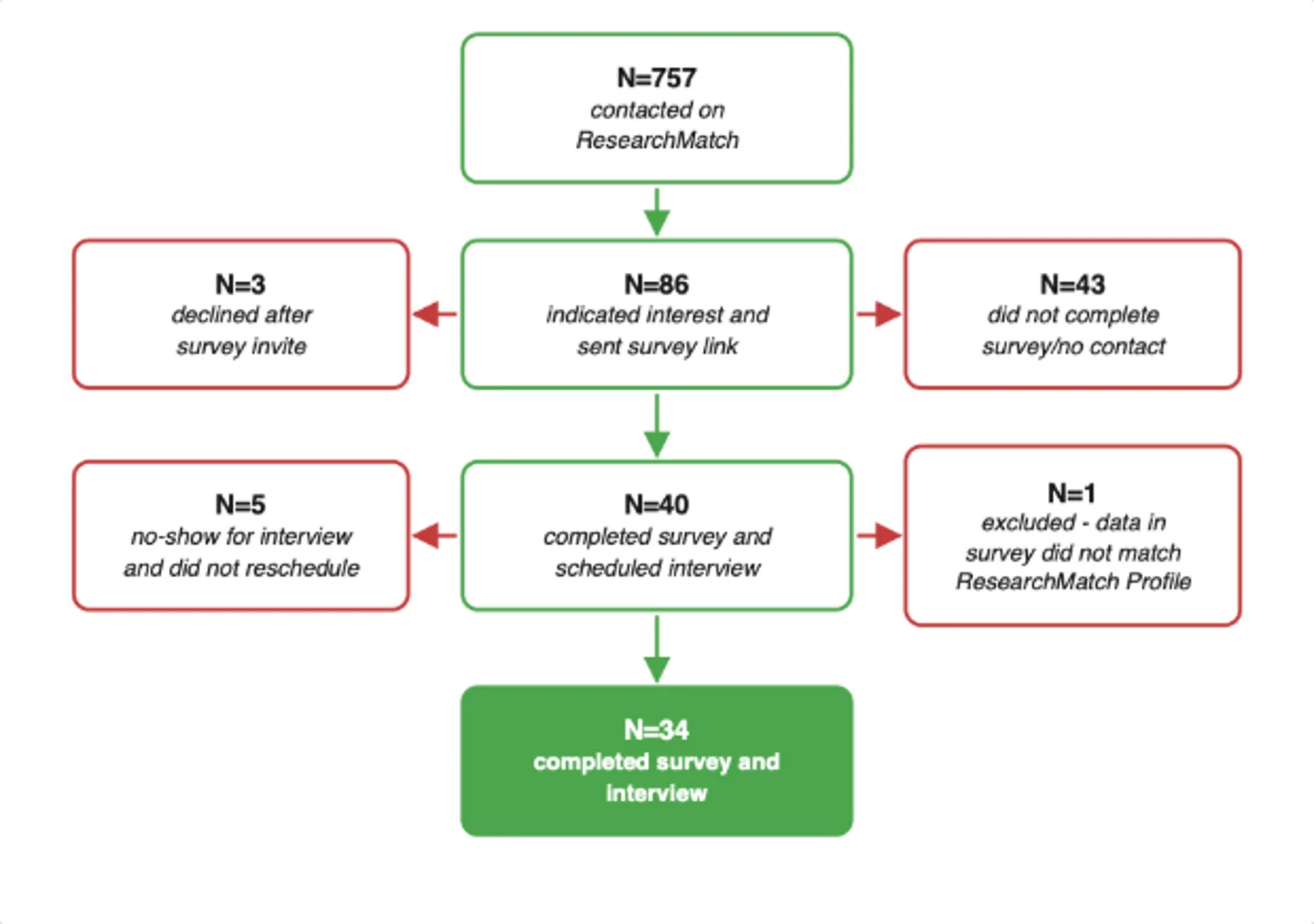
Participant recruitment flow diagram

In total, 34 participants completed the interview and were included in the analytic sample (Fig. 1). The final categorization of participants happened after confirming their details in the interview; while most participants reported having some chronic illness, the participants endorsing being caregivers or social care users were classified as such. Thus, the final categorizations were: 15 (44%) participants with chronic illness, 11 (32%) caregivers, and 8 (24%) social care users (Table 1). Participants ranged in age from 25 to 81 years (mean = 55, SD = 18). The sample was predominantly (71%) female. Most participants (*n* = 27) identified as White and holding at least a bachelor’s degree (*n* = 25). Participants were relatively evenly dispersed across US census regions.

**Table 1:** Participant characteristics

Characteristic	Count	Percent (%)
Average Age, Mean (SD)	55.4 (18.1)	
Age Range, years	25–81	
*Age categories*		
18–34	5	14.7
35–64	14	41.2
≥65	15	44.1
*Gender*		
Male	8	23.5
Female	24	70.6
Non-binary or Other	2	5.9
*Race* ^*a*^		
White	27	79.4
Black or African American	2	5.9
Asian	1	2.9
Mixed Race	2	5.9
Hispanic	3	8.8
*Highest level of education*		
Graduate or professional degree	16	47.1
Bachelor’s degree	9	26.5
Associate degree	6	17.6
Non-college trade or technical school	1	2.9
Some college but no degree	2	5.9
*US census region*		
Northeast	6	17.6
Midwest	11	32.4
South	9	26.5
West	8	23.5
*Inclusion category* ^*b*^		
Chronic Illness	15	44.1
Caregiver	11	32.4
Social care user	8	23.5
*Number of reported chronic conditions*		
1–3	16	47.1
4–7	12	35.3
8–11	6	17.6
*General health rating*		
Very good	2	5.9
Good	13	38.2
Fair	13	38.2
Bad	6	17.6
Very bad	0	0.0
*Caregiving hours per week*		
1–19	8	23.5
20–49	3	8.8
≥50	1	2.9
No response	1	2.9
*Primary daily activity* ^*a*^		
Looking for work	1	2.9
Employed	16	47.1
Retired	13	38.2
On sick leave or disability	5	14.7
Student	4	11.8
Caring for home or family	2	5.9

SD, standard deviation. a Participants could select more than one category. b Participants could select more than one category; however, they were classified into only one category determining the focus of their interview, reflected here.

### Quantitative results

Visual analogue scale (VAS) scores showed minimal variation across constructs: quality of life VAS scores ranged from 25 to 93 (mean = 66.4, SD = 21.5), health VAS scores from 15 to 90 (mean = 61.9, SD = 21.3), and health and wellbeing VAS scores from 15 to 90 (mean = 60.9, SD = 21.8). Relative to the health VAS, the quality of life VAS showed a small effect size (d = 0.25), while the health and wellbeing VAS showed a negligible effect size (d = 0.05). All three VAS versions were strongly correlated (Quality of life and health, *r* = 0.84; health and wellbeing and health, *r* = 0.96; Quality of life and health and wellbeing, *r* = 0.86). The ICC between the three VAS versions was 0.88 (95% CI: 0.78 to 0.93), indicating strong agreement.

### Qualitative results

While all 34 participants contributed quantitative data, there was insufficient time available to discuss the VAS in two interviews (Participant #018 and Participant #021). Therefore, only 32 participants contributed qualitative data. Saturation was reached after 30 interviews. Three inductively derived, overarching themes (interpretations of constructs, recall periods, and preferences) were comprised of 17 major sub-themes (Table 2). Exemplary quotes are attributed by a participant number, age, gender abbreviation (M, F, or none for nonbinary/other gender), and inclusion reason.

### Interpretations of constructs

#### Health VAS

Twenty participants interpreted the health-VAS to indicate an overall perception of their health considering clinical diagnoses and chronic conditions, mostly reflecting their physical condition. Participants considered the presence or absence of illness, or functional limitations (referencing aging, pain, and diagnostic severity), emphasizing traditional, biomedical understandings of health:

*“So health*,* I pretty much only thought about how I’m feeling. Am I sick? How do I feel like*,* physically? How am I on a daily basis?” (#012*,* 46 F*,* social care user)*.

While mental and emotional aspects of health were not as commonly considered, when prompted to consider whether mental health factored into their VAS ratings, participants shared mixed viewpoints:

*“…Probably just physical and not necessarily including mental health…do I have some type of physical pain or some type of illness.” (#030*,* 47 F*,* chronic illness)*.

Approximately 10% of participants were more holistic in their conception of health, considering both physical and mental health. Some tied mental health to their caregiving roles, which often did not impact their physical functioning as much as their mental functioning:

*“Do I have any other acute physical issues…but then I think of mental health*,* and that is ever changing*,* based on those caregiving roles*,* based on the stress and the emotions and everything that comes with that.” (#029*,* 37F*,* caregiver)*.

#### Health and wellbeing VAS

Participants expanded their considerations when interpreting the HWB-VAS to accommodate their conceptions of wellbeing. Participants mostly interpreted wellbeing as a more expansive and integrated construct than health alone, though interpretations were more variable overall. Nine participants expressed that wellbeing emphasized mental and emotional health - including emotions, mood, and psychological outlook - complementing the physical aspects expressed by health alone:

*“…more on just the emotional and kind of holistic wellbeing of my whole person*,* instead of just physical side effects” (#025*,* 37 M*,* chronic illness)*.

Other conceptions of health and wellbeing were broader, reflecting aspects similar to quality of life, such as living environment or life satisfaction. Some participants made a subtle distinction between health representing a health condition, while wellbeing represented an attitude towards that condition and its management:

*“…health and wellbeing is: how well is your condition managed? Okay*,* so my health overall isn’t very good*,* but how is it managed? Between all the external factors*,* I’m able to function at this level.” (#014*,* 72 F*,* chronic illness)*.

Some participants found wellbeing to be a confusing or vague term and had difficulty distinguishing what wellbeing added to the health construct. Participants suggested that health and wellbeing could be redundant:

*“I could not really tell the difference between health and health and wellbeing…[health] you would go see a doctor for*,* whereas health and wellbeing is more of like the absence of illness or sickness. And that just seems like it’s covered with health*,* so that feels like the same.” (#004*,* 25 F*,* caregiver)*.

However, others viewed health and wellbeing to be distinct, complicating ratings when paired in one VAS version. These participants did not believe the constructs were overlapping enough to rate together:

*“Well*,* I’m not starving. I have a roof over my head. I have lots of good things going on where I think my wellbeing is intact. However*,* my health is not so. That’s where I think if you ask one over the other*,* it would probably get different responses” (#033*,* 66 F*,* social care user)*.

Overall, the health and wellbeing VAS was more broadly interpreted to encompass physical, mental, and emotional states, as well as an individual’s coping, functionality, and overall satisfaction with life. However, the conceptual overlap between health and wellbeing posed some challenges. Participants faced difficulties in articulating clear and consistent definitions while considering the differentiation between physical and mental health or narrower conceptions of health and holistic experience.

#### Quality of life VAS

Similarly, participants had broad conceptions of quality of life. Participants often used language like “overall picture” or “big picture” to convey a broader orientation that included health, lifestyle, and economic factors. Three participants also mentioned consideration of “purpose” in their life:

*“Relationships with people*,* family*,* purpose in life - I do a lot of volunteering*,* to me*,* that’s purpose. I have hobbies and book clubs …all of those things go into quality of life. And enough money!” (#016*,* 77F*,* social care user)*.

Two especially common elements included social relationships and finances. Participants described the vital importance of finances and the central role of social connection and a sense of belonging as important components of quality of life:

*“When I think of quality of life*,* I don’t only think of myself*,* but my family*,* my roommate*,* my cat*,* the kind of the housing situation I’m in thinking more along the lines of like*,* financial concerns.” (#025*,* 37 M*,* chronic illness)*.

Seven participants focused mostly on health, more consistent with “health related quality of life”, though this was not presented as a VAS option. However, most did not limit their responses to a clinical or health-oriented perspective, describing quality of life as a comprehensive picture of their lived experience, emphasizing a connection between their perceptions of their health and ability to take care of the health of themselves and their loved ones:

*“Some people would probably tell you I want a sports car and a yacht for a better quality of life. That’s not what I thought of… It’s more health*,* and being in nature*,* and appreciating*,* you know*,* the little things… I’m linking it to her [my mother’s] quality of life… I want to be as accommodating as possible.” (#031*,* 43 M*,* caregiver)*.

Participants also commented that their quality of life could be rated highly despite their poor health or disability, providing qualitative insights which supported the quantitative findings where quality of life ratings were higher than those for health or health and wellbeing:

*“I can be perfectly healthy and lonely*,* bringing my quality of life way back down. Or I can be in a good community*,* have people around me that are supportive*,* so my quality of life can actually be quite okay*,* although my health is poor. I have cancer*,* for instance… but the quality of life*,* because of all the support*,* because of my surrounding*,* is better than that.” (#001*,* 81 F*,* chronic illness)*.

*“But like*,* I’m happy*,* I have really supportive environment. I have a lot of socioeconomic resources. I have hobbies. I have people who value what I contribute to society*,* even though it’s not the traditional things. Like*,* I have meaning in life*,* and I’m someone who would rate my health in the bottom.” (#020*,* 36 F*,* social care user)*.

### Recall periods

#### No recall period

The VAS versions that the participants rated and discussed were initially presented without a recall period. When asked if participants were considering a certain timeframe in their responses, they provided varied answers, ranging from “now” to “the past 10 years”, but usually encompassing several months:

*“I guess I was just thinking about overall - not a specific week or a day. Just overall*,* in general…probably the last couple of months.” (#009*,* 79 F*,* social care user)*.

Three participants explicitly mentioned without prompting that they defaulted to considering the past 7 days, as specified in the EQ-HWB-9 items. Other responses varied based on the construct under consideration, with broader constructs like wellbeing, and especially quality of life, being considered over a longer timeframe:

*“If this question was happening later on in the survey*,* which I think it was*,* and if up until now everything had been based off of the past 7 days*,* then not paying attention to details here pretty much*,* I would just roll through and think that we’re still just talking about [the past 7 days]” (#032*,* 46 M*,* caregiver)*.

*“The last 7 days gives you a snapshot of where you are now. But these quality of life*,* and health*,* and health and wellbeing as an overall deduction or conclusion of what you are*,* not only within the last 7 days*,* but where you are in life now*,* depending on however far back your mental framework wants to take you…No*,* I don’t have a specific timeframe. It’s where I am now and where I’ve been the last 2*,* 3*,* 4 years.” (#011*,* 78 M*,* chronic illness)*.

While some participants were confused by the lack of recall period, others specifically preferred having no recall period so they had the flexibility to consider a recall period that aligned with the course of their illness, disability, or caregiving experience. These participants used the absence of a recall period as an opportunity to reflect in a more contextual and individualized way, adjusting their mental timeframe based on recent life events:

*“I think*,* for health and health and wellbeing*,* I was answering for now*,* in this moment*,* in this last week or two… but quality of life*,* it’s more*,* probably the last year*,* as family members’ health has changed in the last year. So*,* I more look at kind of where this next level started and look back in that period… for now*,* this season has been about a year*,* so I think about this whole year.” (#029*,* 37 F*,* caregiver)*.

#### Today

Interestingly, while many participants used “now” or “the present” to describe their approach to answering the items with no recall period, they did not necessarily mean just “today” in their responses, which was presented to them as an alternative option for the VAS items. Today was interpreted more narrowly. Compared to the flexibility allowed by no recall period, participants felt that restricting the recall period to just “today” made the versions too specific:

*“I was thinking about right now*,* this moment*,* today.” (#007*,* 70 F*,* caregiver)*.

*“I mean*,* then it really narrows the focus. To me. I feel like that [today] changes the question slightly. I mean*,* it doesn’t make it bad. It just definitely would narrow the focus. And it would probably slightly change how I would answer the questions.” (#012*,* 46 F*,* social care user)*.

There was a sentiment that an assessment of “today” may catch respondents on a “bad day” and not provide an accurate overall picture of their health, wellbeing, or especially quality of life. This was especially significant for participants whose health status changed day-to-day:

*“[Today] would make a difference because I have good days and bad days. I probably have more bad days than I do good. Personally*,* I don’t have a whole lot of good days where I’m not worrying to death about everything… So yeah*,* I would have answered it different.” (#005*,* 59 F*,* caregiver)*.

*“Well*,* I mean*,* if you had asked me the day that I got diagnosed with something rather horrific*,* I would probably have put a zero.” (#009*,* 79 F*,* social care user)*.

However, there was variability in a preference for “today” that depended on the construct. Participants also described that even if the item specified today, they would not limit themselves to only considering that one day:

*“No*,* because today is not just today*,* right? It is everything from the past that made my today. (#002*,* 33F*,* chronic illness)*

*“It [today] does [make the question easier to answer]. But I also think of*,* you know*,* what’s led up to today.” (#024*,* 48 F*,* chronic illness)*.

#### Past 7 days

The past 7 days elicited more mixed views. Some felt that 7 days was a good balance between asking about today and not specifying a timeframe – they felt like 7 days was a period they could remember and was long enough to give an overall picture of the construct:

*" I don’t change that much within 7 days*,* like my mood can change significantly*,* but I don’t think the scale would be significantly different. If I’m just having a really good day it would be a little bit different*,* but I don’t think it would be significantly different.” (#017*,* 37*,* social care user)*.

Similar to today, some also felt that 7 days was still too restrictive, especially for broader interpretations of constructs:

*“Quality of life feels like a really big word*,* and I don’t feel like 7 days can accurately encompass all of it. I feel like something like quality of life is a term that required really wanting to think about things like a longer term way than just 7 days.” (#004*,* 25 F*,* caregiver)*.

There was also a similar sense that the timing of the assessment might occur in an abnormal week that would not provide an accurate picture of their overall assessment that the constructs seemed to be inquiring about:

*“My husband’s illness is such that he can have a week or two that’s pretty bad*,* and then he can have weeks where he’s fine. So*,* it’s just for his specific illness*,* seven days sometimes is still too zoomed in*,* I guess.” (#029*,* 37 F*,* caregiver)*.

### Preferences for VAS versions

Overall, across 32 interviews, recall‑period preferences were split, with 15 participants favoring no recall period, 9 participants favoring the past 7 days, and 8 participants favoring today. However, participant preferences were nuanced depending on the construct being assessed. When asked to justify the selection of one (or more) VAS versions to include alongside the EQ-HWB, participants approached their selection from the position of either complementing the EQ-HWB-9 descriptive system or capturing additional information about themselves that may have been lacking from the EQ-HWB-9 items:

*“I would use your health and wellbeing because that covers your physical and mental thoughts… while in quality of life*,* a person might only pick out certain things.” (#005*,* 59F*,* caregiver)*.

*“But in terms of like getting new information*,* I think the quality of life or health and wellbeing*,* which to me and my brain are interchangeable*,* I think those would maybe give you new information.” (#020*,* 36 F*,* social care user)*.

**Table 2:** Thematic summary

Point of inquiry	Theme	Additional representative quotes
Health	Biomedical view of health as physical functioning or absence of illness	*“I guess*,* health being*,* you know*,* fitness and weight and cardio ability*,* different conditions that I’ve been treated for and medicines I’ve been taking….I guess I should also include mental health.” (#024*,* 48 F*,* chronic illness)* *“You know I don’t [think of mental health]. I think honestly I should. But this health strikes me as just the physical side of it. But health*,* I think*,* is a broad term… but whether I should or shouldn’t*,* I don’t really combine the mental aspect.” (#032*,* 46 M*,* caregiver)*
	Broader conceptions of health including mental and emotional factors	*“[Health] is mental wise*,* physical wise*,* emotional wise*,* all that.” (#006*,* 38 F*,* caregiver)* *“[Health is] physical and mental.” (#007*,* 70 F*,* caregiver)* *“…any mental aspect like depression or sadness. [Health] is more about medical conditions*,* whether physical or mental.” (#011*,* 78 M*,* chronic illness)*
Health and wellbeing	Wellbeing adds emotional and psychological factors to health	*“And where you’re at now and then the health and wellbeing one. It seems to broaden the health a little bit more. But also it included that sense of maybe satisfaction or enjoyment in it.” (#008*,* 28 F*,* chronic illness)* *“…my health: how I feel*,* am I sick*,* am I struggling with health conditions? And then wellbeing: I feel like that can be physical or emotional*,* that can be a wider ranging term. So I thought about: How is my wellbeing? Am I happy? Am I satisfied? Am I feeling depressed? Do I have a positive self-image?” (#012*,* 46 F*,* social care user)* *“Well*,* my wellbeing would*,* for me*,* be more mental. I mean I guess that’s why you have two words there*,* I mean I associate health with how it was asked. Health with my physical being and wellbeing with my mental.” (#031*,* 43 M*,* caregiver)* *“Wellbeing is*,* you know*,* your attitude toward your circumstances.” (#034*,* 78 M*,* chronic illness)*
	Hard to define; perceived as overlapping with both health and quality of life	*“My health could be poor*,* but I deal with it well*,* so my wellbeing can actually be better than my health. So to put it together in one is a bit difficult. My health may be poorer than my wellbeing*,* and vice versa… putting both of them in the same to me is kind of ambiguous.” (#001*,* 81 F*,* chronic illness)* *“Yeah*,* this one threw me because I didn’t really know how to define wellbeing. So I didn’t really know how to answer this. Maybe they meant mental wellbeing?” (#029*,* 37 F*,* caregiver)*
	Health and wellbeing are two distinct constructs, making a single VAS item difficult to answer	*“Health is whether you’re sick or you’re not sick. With some kind of an injury or disease*,* or what have you. Something beyond your control. Wellbeing is another for me is a whole ‘nother thing… I don’t think it means health.” (#034*,* 78 M*,* chronic illness)*
Quality of life	Broad, holistic conception including social, economic, and environmental factors	*“Friends*,* making enough money to buy the necessities. Relatively good health. Yeah*,* all that. Quality of life seems to be pretty easy to determine.” (#003*,* 80 F*,* chronic illness)* *“The quality of life is an all comprehensive thought: how’s my health*,* my mental health*,* my social interaction with my spouse and my children and my grandchildren? Can I do the things I want to do? It was very comprehensive*,* taking into consideration what makes life worth living.” (#011*,* 78 M*,* chronic illness)* *“I think of this [quality of life] as happiness and being able to pursue happiness. And that would be limited by money*,* or it would be limited by how you feel*,* or your timing of your medicines… quality of life is so broad… because that really determines your financial situation*,* your housing…” (#014*,* 72 F*,* chronic illness)*
	Includes, but is not limited to, health-related factors	*“Whether or not I can do what I want in a day to day life without any difficulty or challenges with respect to my physical health and my work and family…that was my overall idea of quality of life. You know the overall picture*,* the big picture.” (#002*,* 33 F*,* chronic illness)*
	Importance of social relationships and social connection	*“Okay*,* well*,* my quality of life right now*,* I’m imagining I have food*,* I have a home*,* I have enough money to pay my bills*,* I have my wonderful children*,* grandchildren*,* great-grandchildren*,* friends.” (#009*,* 79 F*,* social care user)* *“That’s what I think about quality of life means. I have the ability to take care of someone who needs me. So I have a good quality of life*,* that’s what I’m thinking. Not emotional*,* not personal…I think of it separately*,* quality of life*,* because I think good quality of life means I have the resources available to take care of my health*,* no matter what goes wrong.” (#013*,* 69 F*,* caregiver)* *“I hate to just say it’s finances*,* but you know*,* because it affects everything. So it’s where you live. Then your home*,* your vacations*,* those things are all. That’s what I look at with quality of life.” (#015*,* 54 F*,* caregiver)*
	Health can be poor while quality of life is still rated highly	*“I would say my quality of life is like not the worst. So I have a job. I’m saving. I have a supportive family. A big part that went into my answer was because of my supports and accommodations…I like really struggle*,* so I had to really balance this external*,* of where I’m at in the world with job*,* work*,* income*,* support systems.” (#008*,* 28 F*,* chronic illness)*
No recall period	Flexible interpretation based on personal relevance	*“I straight up went to when I first started getting that pain in my knee…until today or yesterday…one event or one moment shouldn’t be defining a moment of my quality of life overall*,* and when I say quality of life I think of a broader time range.” (#002*,* 33 F*,* chronic illness)* *“Timeframe really didn’t pop in my head. I suppose if I’m really thinking about it*,* I would say within the past 2 years or less…” (#004*,* 25 F*,* caregiver)* *“Maybe like 10 years. I’ve had a lot of physical changes. That’s when my health issues kind of all started.” (#019*,* 46 F*,* chronic illness)*
	Participants defaulted to thinking about the past 7 days	*“Oh*,* that’s really interesting*,* because health*,* like*,* I kind of thought of the past maybe 2 or 3 months*,* because I’ve been having an especially bad time. And then*,* for quality of life and health and wellbeing*,* I was more thinking about like*,* maybe the last couple of years.” (#020*,* 36 F*,* social care user)*
Today	Too narrow; not aligned with ongoing health experience	*“What if something happens today that impacts my quality of life? And I answer that*,* which may not be what my normal quality of life is.” (#001*,* 81 F*,* chronic illness)* *“[Today] would probably be too limited. You want people to be open about it. And you don’t want to think about today*,* because today they may be fine*,* but last week or overall… no*,* I just like the more open ended type*,* without the word today.” (#011*,* 78 M*,* chronic illness)* *“Quality of life is such a broader term in my head that it doesn’t encompass daily*,* a day*,* or a set point in time. It’s a span*,* you know. And health as well!” (#015*,* 54 F*,* caregiver)*
	Clear and specific snapshot of current state	*“Truthfully*,* you know why I prefer the one day -- today -- because everything else was over the 7 days. I mean it: you’re having to go back and reflect on the whole week*,* which could be a combination of things. Whereas today is more concise… and it’s gonna be more precise. I can say 100% one way or the other.” (#033*,* 66 F*,* social care user)*
7-day recall period	Balanced timeframe capturing recent experiences	*“I think it would be easier. Yeah*,* I mean for me*,* I’m kind of a very reflective person*,* you know. I try to. At the end of Friday. I’ll look back at my week and kind of think things through*,* and think how things went*,* or if things went really poorly*,* what I could have done differently. So yeah*,* it’s kind of like*,* I know for me personally*,* it’s my sort of default.” (#025*,* 37 M*,* chronic illness)*
	Still too short for broader constructs like quality of life	*“I would give it a little broader timeframe than just 7 days. Because*,* say like in a month. I might have 20 bad days and 10 good days. That’s just an example. That’s how I would think of the answer. If it had a broader*,* then you would know I was having more bad days than good days.” (#005*,* 59 F*,* caregiver)* *“No*,* I wouldn’t do that*,* because that doesn’t give you a clear enough picture of what you’re looking for in a person…you need a bigger timeframe than that. A larger scope.” (#011*,* 78 M*,* chronic illness)*
	Participants defaulted to 7 days from prior questions	*“I had been thinking about the past 7 days since that’s what the questions before were asking.” (#031*,* 43 M*,* caregiver)*
Preferred VAS version	Participants justified version preference based on complementarity with or distinctiveness from the EQ-HWB-9 descriptive system	*“I feel like quality of life would be the best choice*,* because I feel like it’s getting at something different. I feel like you’ve already gotten a picture of the person’s health and wellbeing from the nine questions*,* and I feel like quality of life is sort of like that*,* but it’s a different measure of that.” (#012*,* 46 F*,* social care user)* *“If you’re looking for a person’s perception of their being*,* that’s the health and wellbeing. If you’re just looking for that*,* it could kind of sum up those vague things that went on. It kind of gives you what their perspective is when they’re answering those questions [the EQ-HWB-9]*,* and I almost think this might be a 1st question.” (#014*,* 72 F*,* chronic illness)* *“I think it [the EQ-HWB-9] aligns more to the health than to quality of life*,* and I’m kind of just leaving off the health and wellbeing because I didn’t really understand it… I would keep health*,* and I would keep quality of life…but if I did just have to pick one….maybe I would keep quality of life and not health*,* because I feel all those questions [in the EQ-HWB-9] tell you about health.” (#029*,* 37 F*,* caregiver)*

## Discussion

There were mixed interpretations and preferences for each construct and recall period, though several reoccurring interpretations emerged across each variation. A common interpretation of the health VAS was that it indicated clinical diagnoses and chronic conditions, emphasizing mostly physical health over mental and emotional health. Interpretations of the health and wellbeing VAS were mixed overall, with some participants finding wellbeing redundant with health, reflecting the quantitative results that indicate almost the same ratings for the health VAS and health and wellbeing VAS.

These findings diverge from previous research on the EQ VAS: in large observational studies, patients self-rated their health systematically lower using the EQ VAS compared to the EQ-5D index score – which aligned with instruments measuring functional and physical health – while the EQ VAS better aligned with mental health items and patient’s feelings about their health [9, 10]. However, comparisons to previous research on the EQ VAS are imperfect as participant perceptions of a VAS are partially influenced by the preceding items in the descriptive system [8]. It is possible that respondents perceive an associated VAS to either align with the descriptive system or alternatively, serve as a means to indicate more about themselves based on domains perceived to be underrepresented in the descriptive system. Thus, the EQ VAS may be perceived to indicate mental health following the EQ-5D, which mostly encompasses physical and functional health, while a similar health VAS is perceived to indicate physical health following the EQ-HWB-9, which encompasses a broader array of mental and emotional health items.

A recent quantitative study assessing the measurement properties of a health and wellbeing VAS alongside the EQ-HWB-9 in 1,262 respondents from the Hong Kong general population similarly found high agreement and only modest mean score differences between an EQ-HWB VAS and the standard EQ VAS [26]. The authors attributed observed score differences to a combination of factors, including the broader construct label, the longer recall period, and the differential priming effect of preceding descriptive system items from the EQ-5D and EQ-HWB-9 [26]. Notably, in their study, the EQ-HWB VAS also demonstrated marginally better discrimination for wellbeing-relevant subgroups including informal caregivers and those with mental health problems, suggesting that even modest differences in specification of a VAS may carry a meaningful signal for the target populations of the EQ-HWB.

The quality of life VAS evoked a broader assessment encompassing economic situations, social relationships, purpose in life and/or living environments, in addition to health and wellbeing. This holistic interpretation aligns with broader conceptualizations of quality of life that extend beyond health-related domains, offering additional insights about respondents beyond the descriptive system, though potentially out of scope of the EQ-HWB-9. Whether this is an advantage or disadvantage depends on whether developers intend for a VAS to summarize a measure or provide additional information. Participants reported that ratings for quality of life could still be high even if health or health and wellbeing were low, which was aligned with higher average ratings for the quality of life VAS in the quantitative results. In other qualitative literature assessing the EQ VAS, interpretations varied across cultures [11, 27]. The conceptions of a quality of life VAS as focusing on finances and social relationships could be uniquely reflecting American culture and values. These reflections of contextual factors likely vary across cultural settings, making it important to conduct similar qualitative work in international settings.

Regarding recall periods, our findings revealed important nuances in participant preferences and interpretations. When presented with alternative recall period options, most participants preferred having no specified recall period, as this allowed them to consider timeframes most relevant to their lives, for example, recent events or the course of their disease or caregiving responsibilities. Many participants did not recognize the lack of recall period in the VAS versions presented in the quantitative survey; some admitted to assuming that the 7 day recall period extended to the VAS versions from the EQ-HWB-9. Preferences were contingent on the specific combination of construct and recall period, with quality of life widely regarded as requiring a longer timeframe, suggesting that constructs and recall periods should not be selected independently of one another. The advantages of no recall period identified by participants would come at the cost of standardization, as participants’ self-selected timeframes ranged from “today” to “the past ten years”, compromising between-person comparability and highlighting the importance of clear temporal anchoring in item development. Still, items in PROMIS assessing physical functioning do not specify a recall period to accommodate considerations of activities which are not habitual in a daily or weekly timeframe [18]. 

The conceptual ambiguity surrounding wellbeing reflects broader challenges regarding the operationalization of this construct, which can encompass multiple dimensions including life satisfaction, positive affect, and sense of purpose [5]. When paired with health, the perceptions of wellbeing as a mental and emotional component complementing physical health may minimize its definition from traditional perceptions of wellbeing classified as eudaimonic (feeling a sense of purpose in life), hedonic (day-to- day moods and emotions), or evaluative (feeling satisfaction with life as a whole) [28], which better align with our participants’ perceptions of quality of life. Importantly, despite the name of the instrument, the EQ-HWB was not initially conceptualized as a traditional “wellbeing” measure, and the conceptual development framework was not necessarily aiming to align with strict definitions from the wellbeing literature, nor include a summary item [5, 29]. Still, experiences with the EQ VAS and other literature on global assessments of self-rated health suggest that non-specific global ratings may uniquely capture meaningful aspects of health and wellbeing that standardized measures overlook, including for purposes of decision making in HTA [6, 30]. ISPOR devoted a special issue to exploring the concept of “whole health”, a broad, idealized patient-centered construct to incorporate in measures and ultimately value assessment [31, 32, 33]. It is a compelling point for continued discussion how a VAS might best be implemented with the EQ-HWB-9 to better align the instrument with measuring a broader construct than health alone.

Ultimately, the choice of construct and recall period will depend on what is intended to be measured by EuroQol as the instrument developers. This qualitative analysis reveals extensive possible interpretations from the intended population of users of the EQ-HWB-9 and opens several compelling points for discussion, including whether the purpose of the VAS is to summarize or broaden the existing descriptive system. While the current evidence is insufficient to make a concrete recommendation about how to incorporate a VAS with the EQ-HWB-9, it can inform decision making as part of a constellation of other quantitative [26, 34] and qualitative evidence. This exploratory work opens several avenues for future research, including additional larger-scale quantitative studies to examine the measurement properties of VAS variations, international studies to understand cultural variations in construct interpretation, and examining the value of VAS information for clinical decision-making and HTA in the US where QALY-based evaluations face regulatory constraints.

### Study limitations

Several limitations should be acknowledged. The qualitative evidence could be considered hypothesis generating and the sample size was insufficient to quantitatively test any specific hypotheses. Participants required internet access to participate, excluding individuals with limited digital literacy. Additionally, the sample was predominantly white, highly educated, and recruited from a research volunteer registry. The three target populations were not mutually exclusive; all but two identified as experiencing chronic illness and there was overlap in caregivers and social care users, precluding group-specific insights. The concurrent discussion of VAS options may have artificially heightened distinctions between constructs and recall periods, which might be less salient in real-world administration of the EQ-HWB-9. Anchor wording, held constant to isolate the effects of construct and recall period, is a third design dimension which was not evaluated here; the “best/worst you can imagine” anchors originate in the valuation context in which the EQ VAS was developed [7] and warrant reevaluation if a VAS is intended as a self-reported global assessment rather than a prime for valuation. The interview order was not completely standardized, so the discussion of the VAS items occurred before or after the discussion of the EQ-HWB-9 items, including select items from the long-form EQ-HWB. While no differences in responses were noted based on the interview order, a more pronounced framing effect could have been introduced in participants who first discussed the EQ-HWB-9 items in depth prior to discussing the VAS. Ultimately, VAS items should be tested both quantitatively and qualitatively as they would appear in-situ within the EQ-HWB-9 instrument. Finally, the cross-sectional design precluded assessment of how VAS interpretations might evolve with repeated use or changing health states; this was a point of confusion for participants in interpreting the recall periods as they were not considering repeated, longitudinal administration of the EQ-HWB-9 and VAS.

## Conclusions

This mixed-methods study provides valuable insights into how US participants interpret and engage with different VAS constructs and recall periods for potential inclusion alongside the EQ-HWB-9, meant to promote continued conversations within the EuroQol group. It revealed complex interpretive processes underlying VAS versions and highlights important considerations to meaningfully implement one or more VAS items with the EQ-HWB-9. The decision of why and how to include a VAS alongside the EQ-HWB-9 is layered and should be guided by global quantitative and qualitative evidence that addresses technical considerations of measurement properties and the intended uses of the EQ-HWB-9 in research, clinical, HTA, and policy contexts.

## Supplementary Information

Below is the link to the electronic supplementary material.

Supplementary Material 1

## Data Availability

No datasets were generated or analysed during the current study.
