# Supplementary material for: Exploration of visual analogue scale versions with the EQ-HWB-9 to measure health, social care, and carer-related quality of life: a VASt landscape
Source: Qual Life Res. 2026 Aug 7;35(9):258. doi: 10.1007/s11136-026-04369-8 (PMC13451295; doi:10.1007/s11136-026-04369-8)
Supplement: Supplementary file 1 [file 11136_2026_4369_MOESM1_ESM.docx]

**Supplementary Material: Comparison of Nine VAS Versions**

Note that in the online survey, only one VAS version from the first row was presented at a time, in a random order. In the interview, VAS versions were covered and uncovered during the conversation, so a maximum of three versions (one complete row) was shown at one time.
